# A Novel Method for Estimating Myocardial Strain: Assessment of Deformation Tracking Against Reference Magnetic Resonance Methods in Healthy Volunteers

**DOI:** 10.1038/srep38774

**Published:** 2016-12-12

**Authors:** Kenneth Mangion, Hao Gao, Christie McComb, David Carrick, Guillaume Clerfond, Xiaodong Zhong, Xiaoyu Luo, Caroline E. Haig, Colin Berry

**Affiliations:** 1 BHF Glasgow Cardiovascular Research Centre, University of Glasgow, UK; 2 West of Scotland Heart and Lung Centre, Golden Jubilee National Hospital, Clydebank, UK; 3 School of Mathematics and Statistics, University of Glasgow, UK; 4 Clinical Physics, NHS Greater Glasgow and Clyde, Glasgow, UK; 5 MR R&D Collaborations, Siemens Healthcare, Atlanta, GA, USA; 6 Robertson Centre for Biostatistics, University of Glasgow, UK

## Abstract

We developed a novel method for tracking myocardial deformation using cardiac magnetic resonance (CMR) cine imaging. We hypothesised that circumferential strain using deformation-tracking has comparable diagnostic performance to a validated method (Displacement Encoding with Stimulated Echoes- DENSE) and potentially diagnostically superior to an established cine-strain method (feature-tracking). 81 healthy adults (44.6 ± 17.7 years old, 47% male), without any history of cardiovascular disease, underwent CMR at 1.5 T including cine, DENSE, and late gadolinium enhancement in subjects >45 years. Acquisitions were divided into 6 segments, and global and segmental peak circumferential strain were derived and analysed by age and sex. Peak circumferential strain differed between the 3 groups (DENSE: −19.4 ± 4.8%; deformation-tracking: −16.8 ± 2.4%; feature-tracking: −28.7 ± 4.8%) (ANOVA with Tukey post-hoc, F-value 279.93, p < 0.01). DENSE and deformation-tracking had better reproducibility than feature-tracking. Intra-class correlation co-efficient was >0.90. Larger magnitudes of strain were detected in women using deformation-tracking and DENSE, but not feature-tracking. Compared with a reference method (DENSE), deformation-tracking using cine imaging has similar diagnostic performance for circumferential strain assessment in healthy individuals. Deformation-tracking could potentially obviate the need for bespoke strain sequences, reducing scanning time and is more reproducible than feature-tracking.

The standard method for describing left ventricular (LV) pump function, the LV ejection fraction (LVEF), is based on the displacement of the endocardial borders during the cardiac cycle, and myocardial contractility is not directly assessed. Although echocardiography is the widely-used standard of care for imaging cardiac structure and function, cardiovascular magnetic resonance (CMR) has superior signal-to-noise ratio and endocardial border definition, and superior reproducibility^1^. In addition, echocardiography is limited by acoustic shadows and body habitus. LVEF derived by echocardiography is user-dependent and potentially imprecise^2^,^3^,^4^.

Myocardial strain is defined as the deformation of an object relative to its original length. Strain reflects myocardial deformation and is more closely linked with myocyte metabolism and contractility than LVEF^5^. Fibre shortening is reflected by a negative value for circumferential and longitudinal strain, whereas radial strain is positive, since LV thickening occurs with contraction. Strain can be estimated with echocardiography, cardiac magnetic resonance imaging or computed tomography (CT)^6^, and described globally or segmented for myocardial regions. A validated method for estimation of myocardial strain involves displacement encoding with stimulated echoes (DENSE). This technique has been validated using phantoms^7^,^8^,^9^, and myocardial tagging^10^,^11^,^12^,^13^,^14^.

Feature-tracking is a technique which derives strain from routinely acquired cine sequences, avoiding additional breath-hold imaging acquisitions that would be required with a bespoke strain method, such as DENSE, therefore shortening the duration of the CMR examination. Strain is derived by tracking features of interest along the endo- and epi-cardial borders as well as from columns of pixels radiating out from the endocardium^15^. Feature-tracking derived strain has been described as having larger magnitudes when compared to strain derived from reference methods, such as myocardial tagging, in healthy volunteers^16^, and in patients with cardiovascular disorders^17^,^18^,^19^. Displacement at the tissue-blood pool interface may be subtly greater than within the myocardial tissue, and this discrepancy may explain the potential inaccuracy of feature-tracking^20^.

Biomathematicians in our group (H.G., X.L.) have developed a method of estimating myocardial strain designed to overcome some of the theoretical limitations of feature-tracking, which uses local features, by estimating pixel-wise strain for myocardial deformation incorporating both local and global myocardial features from cine images. Deformation-tracking^21^ is based on an in-house intensity-based b-spline deformable registration method^22^. Our prelimary study^21^ and other studies^23^,^24^ have demonstrated that deformable image registration methods could be an alternative way to quantify regional LV deformation.

Circumferential strain (Ecc) is a key function inherent in myocardial contractility. The aim of our study was to compare two different cine-strain methods with a reference method of strain (DENSE). Since deformation-tracking derives strain from the entire myocardial region of interest, we hypothesised that circumferential strain derived with this method, would match strain derived from DENSE more closely than strain derived using feature-traking software. To this end, we enrolled a reasonably large group of healthy volunteers across a broad age range with no upper limit.

## Results

### Characteristics of the Study Participants

89 subjects underwent cardiac MRI at 1.5 T. Of these, 81 patients had short-axis views acquired with DENSE. The characteristics of these participants (n = 81) and their LV mass and function are presented in Table 1.

#### Quality Assessment

Image quality was higher for the cine scans than the DENSE acquisitions (Table 2). DENSE and deformation-tracking had better reproducibility than feature-tracking, (Table 3), and the intra-class correlation co-efficient was >0.90 for all analyses. Feature-tracking derived strain had larger standard deviations and wider limits of agreement.

The mean time (standard deviation) taken to analyse a short-axis slice was 105 ± 11 s for deformation-tracking, 53 ± 1 s for DENSE, and 76 ± 1 s for feature-tracking (ANOVA p < 0.009).

#### Strain results

**Figure 1:**
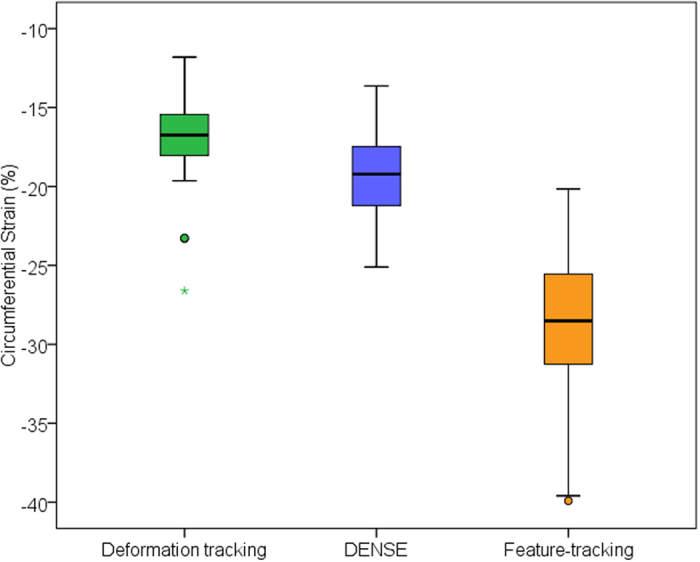
Peak circumferential strain assessed in 81 healthy volunteers using deformation-tracking, feature tracking and DENSE. Outliers are depicted by circles.

Peak circumferential strain was lower with tissue deformation-tracking than with DENSE (deformation-tracking: −16.8 ± 2.4%, variance 4.7, DENSE: −19.4 ± 4.8%, variance 6.4). Feature-tracking derived strain was higher in magnitude than with the other two methods (−28.7 ± 4.8%, variance 23.2). Peak strain differed between the three methods (ANOVA with Tukey post-hoc, F-value 279.93, p < 0.01) (Fig. 1). Regional differences in strain were also observed with all three methods. Segmental data with feature-tracking had higher standard deviations when compared against DENSE and deformation-tracking (Table 4).

#### Sex differences in LV strain

Global circumferential strains were greater in magnitude in women than in men with all three methods (Table 4) though the differences were not detected to be significantly different with feature-tracking, reflecting a consistent sex-related difference in strain.

#### Age differences in LV strain

There was no correlation between age and strain as revealed by DENSE (Pearson coefficient = 0.45, p = 0.70); Weak correlations were revealed by deformation-tracking (Pearson coefficient = 0.249, p = 0.025) and feature-tracking (Pearson coefficient = 0.379, p < 0.01). There was no difference in peak circumferential strain with age with DENSE and with deformation-tracking, after accounting for sex in a multivariate regression model. Using feature-tracking, there was a significant relationship with age, but not sex (Table 5).

## Discussion

We have assessed the diagnostic performance of a novel deformation-tracking method for the assessment of circumferential strain across the full thickness of the myocardium. The method differs from feature-tracking which is mainly restricted to strain estimation from border deformation. We assessed both methods in a group of healthy volunteers with a broad age range and reasonably balanced sex distribution, and compared the strain values from these methods against strain data derived from a reference method (DENSE). Both deformation-tracking and feature-tracking enable strain estimation from standard cine scans.

We found that the shortest analysis time was with DENSE, and the longest time was with deformation-tracking, but overall the analysis times were broadly similar (<1.5 minutes) for all 3 methods. In a small group of patients with acute myocardial infarction, feature-tracking strain analysis was shown to be more reproducible than tagging analysis with a shorter analysis time^17^.

**Figure 2:**
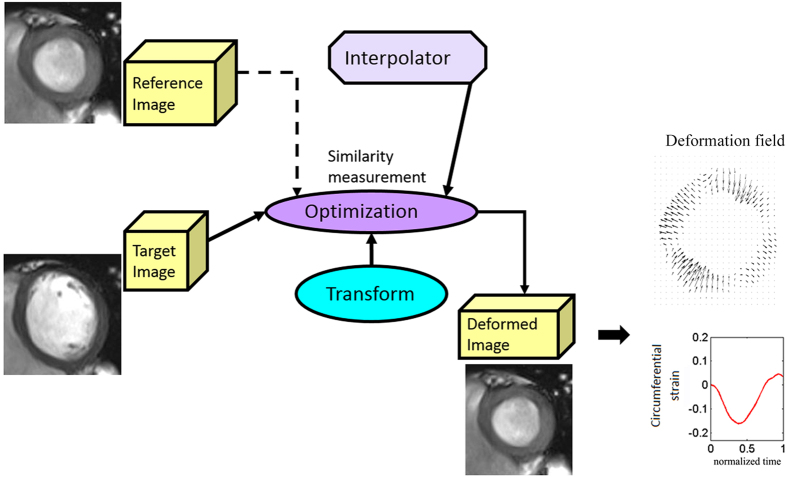
Cine-strain estimation with in-house developed tissue deformation-tracking software. Schematic illustration of deforming a target image to a reference image using a b-spline deformable registration method. Myocardial strain is calculated from the estimated deformation fields. The similarity measurement is defined as the sum of squared differences in pixel intensity between the two images, the deformation transformation between the target and the reference images is modeled with uniform cubic b-splines.

The peak circumferential strain derived from tissue deformation-tracking was lower than DENSE, whereas peak strain was higher with feature-tracking. Our results are consistent with those from previous studies^16^,^17^,^18^, in which feature-tracking over-estimated systolic strain when compared with reference data derived from myocardial tagging which is a dedicated strain imaging technique (Fig. 2, Table 4). On the other hand, some studies have reported similar circumferential strain values between feature-tracking and tagging in patients with Duchenne’s muscular dystrophy^19^, and in patients with acute myocardial infarction^17^ were both endo- and epicardial derived feature-tracking strains were averaged though the limits of agreement were wider than with tagging.

Deformation-tracking identified a sex difference in strain as previously reported using DENSE^25^, i.e. that females may generate larger magnitudes of circumferential strain. In this study, feature-tracking derived strain revealed larger magnitudes of strain in females, which were not statistically significant. This result is in keeping with other studies using feature-tracking to look at healthy volunteers. Augustine^16^ (n = 145, 37% male) and Taylor^26^ (n = 100, 50% male) reported that circumferential strain was not associated with sex, whilst André^27^ (n = 150, 50% male) identified a statistically significant difference in circumferential strain between the sexes, with females having larger magnitudes of strain.

Due to sex-related association with peak strains as revealed by DENSE and deformation-tracking, we used a multivariate regression model to account for the influence of sex on strain, there was no association with age for DENSE and for deformation-tracking. Feature-tracking identified a relationship of strain with age. Whilst the best-fit regression line might be statistically significant, the spread (or variance) of the results is wider with feature-tracking than with deformation-tracking or DENSE, possibly due to the over-estimation of values by this technique, and the clinical significance of this result is uncertain. Looking to the literature, André^27^ using feature-tracking, Oxenham^28^ using tagging, and Neizel^29^ using strain-encoded CMR did not identify any association between age and strain. On the other hand Taylor^26^ reported an age related increase in circumferential strain, but no information was provided on the potential associations with sex.

There was marked heterogeneity in segmental values in feature-tracking and deformation-tracking, in keeping with that observed in studies with large numbers of healthy volunteers^16^. Segmental circumferential strain with feature-tracking had higher standard deviations than segmental strain acquired with DENSE or deformation-tracking, though segmental differences in healthy volunteers utilising feature-tracking strain have been previously described^27^.

There is controversy in the literature whether segmental feature-tracking derived strain is ready for clinical utility, as it has lower intra-class correlation co-efficients as compared to tagging, and because of high inter-study variability^20^,^30^. On the other hand studies have shown that segmental circumferential strain has utility in predicting segmental recovery post infarction^31^,^32^; as well as being correlated with infarct size^17^. Further, the analysis and interpretation of segmental strain data should account for repeated sampling, since strain within neighbouring segments is not independent.

A limitation of this study is that only mid-LV circumferential strain was assessed.

Tissue deformation-tracking performed well when compared with a reference method. The advantage of utilising a cine-strain derived method is that this would obviate the need for bespoke strain sequences being acquired, limiting the total duration of an CMR scan, and making strain more accessible in the clinical setting. Future research should assess whether deformation-tracking can accurately identify pathology such as infarct scar, and whether strain derived from deformation-tracking predicts prognosis.

## Methods

### Study Population

The UK Research Ethics Service (ethics reference 11/AL/0190) approved the study and all of the participants provided written informed consent. Healthy volunteers aged at least 18 years with no prior medical history (including cardiovascular health problems, medication or systemic illness) were invited to participate by placing advertisements in public buildings (e.g. hospital, university). The other exclusion criteria included standard contraindications to MR (e.g. metallic implants and metallic foreign body) and known or suspected pregnancy. A 12-lead electrocardiogram (ECG) was obtained in all subjects and a normal ECG was an eligibility requirement. Patient characteristics were recorded and body surface area was calculated with the Dubois formula.

### MR Acquisition

Participants underwent CMR at 1.5 T (MAGNETOM Avanto, Siemens Healthcare, Erlangen, Germany) located in the Radiology Department of our hospital, using an anterior phased-array body coil (12-element) and a posterior phased-array spine coil (24-element). MR scanning was performed in line with current international guidelines^33^.

### MR protocol

LV dimensions were assessed using balanced steady state free precession (b-SSFP) cinematographic breath-hold sequences. Typical imaging parameters are as shown in Table 6. The heart was imaged in multiple parallel short-axis planes 7-mm thick separated by 3-mm gaps, as well as in the 2-chamber, 3-chamber, and 4-chamber long-axis views.

A two-dimensional (2D) echo planar imaging (EPI) DENSE sequence (work-in-progress sequence 611, Siemens Healthcare, Erlangen, Germany) was used to acquire mid-ventricular short-axis and 4-chamber long-axis views. Typical imaging parameters are shown in Table 6. Through-plane dephasing and 2-point complementary spatial modulation of magnetization (CSPAMM) were used for artefact suppression during DENSE acquisition^34^. Fat suppression was carried out using a fast water excitation option provided by the vendor. The readout and phase-encoding direction of displacement were acquired in a single breath-hold.

Participants over 45 years of age had a blood chemistry test and if the estimated glomerular filtration rate (eGFR) was >30 mls/min/1.73 m^2^ gadolinium contrast was administered (0.15 mmol/kg per bolus of gadolinium diethyltriaminepenta -acetic acid (Gd-DTPA, Magnevist, Bayer Healthcare). Late gadolinium enhancement imaging covering the entire LV was performed 10–15 minutes after intravenous contrast agent administration using a segmented phase-sensitive inversion recovery (PSIR) turbo fast low-angle shot pulse sequence.

### Image Analysis

Image analysis is as previously described^25^. The imaging datasets were anonymised to ensure that the imaging analysts were blinded to all other data. The strain data for each method were analysed separately in order to minimise observer bias. The data were co-ordinated by G.C. and C.H.

DENSE data were analysed using the CIM_DENSE2D software (University of Auckland, New Zealand)^35^ as previously described^25^. K.M and G.C. carried out DENSE image quality and artefact scoring and X.Z. acted as a blinded independent adjudicator when there was discordance. Cine image quality and artefact scoring was carried out by K.M. and C.M. and C.B. acted as a blinded independent adjudicator when there was discordance. The assessments involved using a Likert scale based scoring system developed by C.M.^25^.

Diogenes feature-tracking software (TomTec Imaging Systems, Germany) was used to quantify strain from cine images. The same operator (K.M.) derived strain following a standard protocol in line with the software manufacturer’s instructions^16^,^19^.

Deformation-tracking strain was calculated by the same operator (K.M.) using in-house developed code by H.G. in Matlab (Mathworks, Natick, US), in line with a standard, automated protocol that minimised variability and bias (H.G.)^21^. In the deformation-tracking approach^21^ (Fig. 2), an intensity-based b-spline deformable registration method^22^ was implemented to deform one target image into a reference image by minimizing the sum of squared differences of the two images, the deformation fields were represented by cubic b-spline basis functions, and further smoothed by introducing a regularized term in the minimization procedure. The deformation gradient **F** inside the LV wall was defined as 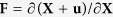, **u** is the tracked deformation field inside the LV wall, **X** denotes the reference position of the LV wall. Finally the 2D myocardial strain tensor, defined as 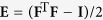, was summarized for each segment, where I is the identity matrix. Details of deformation-tracking approach can be found in our preliminary study^21^.

The results were saved as text files, and then the data were extracted using in-house code written in Matlab (Mathworks, Natick, US) by C.M. to minimize transcription errors.

The LGE images were reviewed by 2 experienced imaging cardiologists (D.C. and C.B.). The presence of LGE would imply sub-clinical or incidental cardiac disease such that the participant would then ineligible for continuing in the study.

### Quality assessment

To minimise observer bias, 20 DENSE and 20 cine images were identified at random by G.C., and coded using a different code sequence to the main dataset. These 20 DENSE and 20 cine images were re-analysed 2 weeks later, by the same operator (K.M.) and by a 2^nd^ expert operator (C.M. for DENSE, feature-tracking; H.G. for deformation-tracking). The time taken to analyse 20 data sets was measured for each of the operators and averaged. The study identifiers were released by G.C. once the analysis was carried out by K.M., C.M. and H.G.

### Statistical Analysis

The statistical analyses were performed using SPSS software (SPSS Inc, Chicago, IL, USA, version 22). Normality was tested using the Shapiro-Wilk test. Continuous variables were expressed as mean ± standard deviation (SD). For comparison of two or more normally distributed variables, Student’s t-test and ANOVA with Tukey post-hoc analysis, or multivariate regression analysis were used. A p-value of <0.05 was considered statistically significant.

## Additional Information

**How to cite this article:** Mangion, K. *et al*. A Novel Method for Estimating Myocardial Strain: Assessment of Deformation Tracking Against Reference Magnetic Resonance Methods in Healthy Volunteers. *Sci. Rep.* **6**, 38774; doi: 10.1038/srep38774 (2016).

**Publisher's note:** Springer Nature remains neutral with regard to jurisdictional claims in published maps and institutional affiliations.

## Figures and Tables

**Table 1:** Demographics of the healthy volunteers (n = 81).

Demographic	
Age (years)*	44.6 ± 17.7
Sex (Male) n (%)	39 (48)
Height (cm)*	170 ± 9
Weight (kg)*	75.8 ± 15.1
Body mass index, kg m^−2^*	26 ± 4
Body surface area (m^2^)*	1.86 ± 0.21
LVEF (%)	63.6 ± 5.2
LVEDV index (mL/m^2^)*	70.1 ± 11.3
LVESV index (mL/m^2^)*	25.8 ± 6.6
LV mass index (g/m^2^)*	40.4 ± 9.7
Myocardial segments analysed	486

LVEF: Left ventricle ejection fraction; LVESV: Left ventricle end-diastolic volume; LVESV: Left ventricle end-systolic volume.

^*^Mean ± standard deviation.

**Table 2:** Image quality and artefact scoring.

Image quality	DENSE (n = 81)	Cine scans (n = 81)
High quality^1^	76 (94%)	81 (100%)
Adequate quality^2^	5 (6%)	0 (0%)
Non-diagnostic^3^ Number of images with	0 (0%)	0 (0%)
motion artefact Number of images with	2 (2%)	0 (0%)
field effect artefact	1 (1%)	0 (0%)
Segments excluded	0 (0%)	0 (0%)

^1^High quality: Well defined endo- and epicardial borders at end-systole. No ghosting due to patient breathing. No flow artefact.

^2^Adequate quality: One or more of the following were present: Slight blurring of endo- and epi-cardial borders at end-systole. Slight artefact due to flow within blood pool but not affecting myocardium. Slight ghosting due to patient breathing.

^3^Non-diagnostic: One or more of the following are present: Loss of endo- and epi-cardial borders at end-systole; ghosting due to respiration.

**Table 3:** Reproducibility of analysis.

Variable	Mean bias ± SD (%)	95% Limits of agreement	p-value	ICC	Correlation R	p-value
DENSE-intra	−0.10 ± 0.50	−1.06 to 0.80	<0.01	0.95	0.934	<0.01
DENSE-inter	0.05 ± 0.60	−1.01 to 1.12	0.53	0.94	0.906	<0.01
Deformation-tracking-intra	−0.07 ± 0.27	−0.61 to 0.48	0.33	0.97	0.960	<0.01
Deformation-tracking-inter	−0.11 ± 0.34	−0.79 to 0.58	<0.01	0.95	0.953	<0.01
Feature-tracking-intra	−0.14 ± 0.89	−1.92 to 1.64	0.49	0.97	0.978	<0.01
Feature-tracking-inter	0.40 ± 1.21	−2.02 to 2.82	0.15	0.96	0.955	<0.01

ICC: intra-class correlation co-efficient. SD: standard deviation. A sample size of 20 images was taken per variable. The intra-class correlation coefficient is above 0.85 for all of them.

**Table 4:** Circumferential strain utilising DENSE, deformation-tracking and feature-tracking.

Parameter E_cc_*	DENSE			Deformation-tracking			Feature-Tracking		
	Male (n = 39)	Female (n = 42)	p-value	Male (n = 39)	Female (n = 42)	p-value	Male (n = 39)	Female (n = 42)	p-value
Global (%)	−18.7 ± 2.2	−20.1 ± 2.7	0.014	−16.0 ± 1.7	−17.5 ± 2.7	0.005	−28.0 ± 4.8	−29.2 ± 4.8	0.262
Anterior (%)	−20.3 ± 3.5	−21.6 ± 4.2	0.153	−14.2 ± 3.8	−16.1 ± 3.8	0.053	−29.6 ± 10.1	−28.1 ± 8.9	0.501
Antero-septal (%)	−18.2 ± 3.7	−17.9 ± 3.3	0.667	−17.9 ± 3.9	−20.2 ± 3.8	0.009	−24.8 ± 9.0	−26.9 ± 12.1	0.388
Infero-septal (%)	−16.0 ± 3.6	−17.4 ± 3.0	0.055	−19.4 ± 3.3	−20.6 ± 2.1	0.179	−23.2 ± 8.8	22.9 ± 11.1	0.891
Inferior (%)	−18.5 ± 3.3	−20.8 ± 3.6	0.003	−15.3 ± 2.2	−16.4 ± 3.8	0.141	−26.9 ± 6.1	−24.5 ± 7.6	0.139
Infero-lateral (%)	−21.1 ± 2.9	−22.7 ± 3.7	0.030	−18.9 ± 3.1	−19.7 ± 3.8	0.005	−26.2 ± 7.6	−30.9 ± 9.5	0.019
Antero-lateral (%)	−20.9 ± 3.2	−22.3 ± 3.2	0.047	−14.0 ± 3.2	−14.7 ± 3.1	0.501	−22.5 ± 9.0	026.0 ± 7.7	0.72

Mean (±Standard deviation); E_cc_: Circumferential strain.

**Table 5:** Significance of relationship of strain with age and sex at 1.5 T.

E_cc_	DENSE	Deformation-tracking	Feature-tracking
P value for regression	0.046	0.004	<0.001
Co-efficient for age	−0.005 (−0.036, 0.025)	−0.029 (−0.054, −0.004)	−0.100 (−0.155, −0.450)
P value for age	0.733	0.059	0.001
Co-efficient for sex	1.370 (0.277, 2.463)	−0.029 (−0.054, −0.004)	0.994 (−0.761, 3.197)
P value for sex	0.015	0.006	0.224

E_cc_: Circumferential strain.

**Table 6:** Typical MRI imaging parameters at 1.5 T.

**b-SSFP**	**1.5 Tesla**
TR (ms)	3.3
TE (ms)	1.2
FoV (mm)	340
Flip Angle (degree)	80
Slice Thickness (mm)	7
Resolution (mm)	180 × 256
Bandwidth (Hz/pixel)	930
**DENSE**	**1.5 T**
TR (ms)	32.5
TE (ms)	7.97
FOV (mm)	360
Voxel size (mm)	3.2 × 3.2 × 8
Flip angle (degree)	20
Bandwidth (Hz/pixel)	1207
Displacement Encoding Frequency (π/mm)	0.2
Triggers per breathold	8
EPI factor	8
segments per cardiac frame	16
Shimming method	automatic

TR: repetition time (ms); TE: echo time (ms); FoV: field of view (mm).
